# Dual Roles of Prostaglandin E2 (PGE2) in Bone Remodeling and Pain Management: Bridging the Gap in Osteoarthritis Research

**DOI:** 10.1155/mi/8882429

**Published:** 2025-07-26

**Authors:** Yulian Zhang, Wenzhi Wu, Zhuo Chen

**Affiliations:** School of Stomatology, Zhejiang Provincial Clinical Research Center for Oral Diseases, Key Laboratory of Oral Biomedical Research of Zhejiang Province, Cancer Center of Zhejiang University, Engineering Research Center of Oral Biomaterials and Devices of Zhejiang Province, Stomatology Hospital, Zhejiang University School of Medicine, Hangzhou 310000, China

**Keywords:** bone homeostasis, osteoarthritis, pain, prostaglandin E2

## Abstract

Osteoarthritis (OA) is a bone disease mainly treated with nonsteroidal anti-inflammatory drugs (NSAIDs) to relieve pain. However, the exact mechanisms underlying this disease remain elusive, which creates an attractive opportunity to explore the mechanisms and provide intentional treatments for OA. In this narrative review, we selected articles discussing advancements and applications of PGE2 to OA biology and pathology and discussed how PGE2 reacts during OA-associated pain, the resulting bone structural alterations, and the potential drugs or treatments for patients with OA. We aimed to summarize the accumulating evidence suggesting that prostaglandin E2 (PGE2) plays an important role in the central sensitization of OA-related pain, elucidating the precise mechanisms underlying the pain relief effects of NSAIDs. Additionally, we interpreted the potential mechanisms by which PGE2 influences bone repair and regeneration at different stages of bone remodeling in OA progression, which raises concerns regarding the side effects of NSAIDs in bone remodeling during disease progression. Finally, we discussed the potential therapeutic strategies for different stages of OA based on available evidence. This review focused on the newly found evidence for the novel functions of PGE2 in central sensitization and bone remodeling and provides possible future directions for the treatment of OA.

## 1. Introduction

Osteoarthritis (OA) is a globally prevalent joint disease whose pathophysiology involves the entire joint. It is characterized by inflammation in the cartilage and synovium, leading to articular cartilage degeneration, osteophyte formation, and sclerosis of the subchondral bone [1]. Patients commonly suffer from repeated and aggravated joint pain and swelling, limited activity, and deformity, resulting in reduced quality of life and physical function.

Pain, the main symptom of OA, is the main reason patients seek medical help. Numerous studies have focused on the pathways associated with pain, such as the Wnt/β-catenin signaling pathway, nerve growth factor/tropomyosin receptor kinase A, calcitonin gene-related peptide (CGRP), and C–C motif chemokine ligands 2/chemokine receptor 2. Prostaglandin E2 (PGE2), a lipid signaling molecule involved in pain and inflammation, is gaining attention for its multifunctionality [2–6]. To the best of our knowledge, PGE2 acts on four kinds of E prostanoid rhodopsin-like G-protein coupled receptors (EP1–4), particularly EP4, which is highly expressed in the bone and has an important regulatory role in bone metabolism. Currently, topical or oral nonsteroidal anti-inflammatory drugs (NSAIDs) are the first-line pharmacotherapies used to alleviate OA-related pain [7]. They inhibit the activity of cyclooxygenase (COX) enzymes, one of which is COX2, the rate-limiting enzyme in prostaglandin biosynthesis, thus lowering the levels of PGE2. Additionally, PGE2 interacts with other pain mediators, such as the transient receptor potential vanilloid-1 (TRPV1) channel, a receptor of capsaicin. TRPV1 is an integrator of diverse painful stimuli and is involved in nociceptor sensitization and various pain states. Although previous systematic reviews have reported the effectiveness of NSAIDs in pain treatment, the exact mechanisms underlying OA pain remain unclear.

In addition to the uncertainty regarding the mechanisms of pain in OA, the use of NSAIDs raises concerns regarding their side effects. NSAIDs have been reported to cause severe gastrointestinal [8, 9], cardiovascular [10–12], hepatic [13, 14], renal [15, 16], cerebral [17, 18], and pulmonary [19] complications. Notably, several studies have shown that using NSAIDs influences the self-regeneration and fracture-healing processes of the bone. For example, low doses of aspirin have been found to partially block osteoclast activity [20, 21] and differentiation as well as rescue bone marrow mesenchymal stem cell (MSC) function [22–24], while high doses of aspirin, diclofenac, or indomethacin have multiple effects on osteoblast and osteoclast activities [25–27]. Because bone remodeling can be modified by NSAIDs, it has also been thought of as one of the main pathological alterations in OA. In the early stage of OA, the subchondral bone undergoes resorption, causing a low bone density, which may induce articular alteration since the bone undertakes less mechanical load. In the terminal stage of OA, the subchondral bone suffers from osteosclerosis and osteophyte formation, which causes severe pain and deformity. Thus, the mechanisms by which NSAIDs influence bone remodeling during OA progression are of great importance and remain to be elucidated.

In this review, we highlighted PGE2 as a potential therapeutic target based on recent treatments and studies, and summarized the secretory sources of PGE2. Next, we comprehensively introduced the mechanisms by which PGE2 stimulates pain and influences bone homeostasis in OA. Finally, we discussed possible alternative therapeutic strategies for OA at different stages of the disease, which are crucial for further research and therapy.

## 2. PGE2 Expression Is Increased From Multiple Sources During OA

PGE2, the main member of the prostaglandin family, is produced from the 20-carbon essential fatty acid arachidonic acid, which is cleaved from different biomembranes by specific phospholipases, of which COX2 is considered the key enzyme. NSAIDs are strongly recommended as therapeutic drugs [14] for its function to inhibit COX2 activity and PGE2 synthesis. The validated effectiveness of PGE2 in relieving OA pain indicates that it acts as a target for OA therapy.

In addition, NSAIDs and COX2 inhibitors have significant analgesic effects on OA and can improve joint function [28–30]. Moreover, PGE2 levels have been reported to increase due to damaged articular cartilage during OA [31], indicating that PGE2 plays a vital role in OA progression. However, the exact source of this potential target has not been specifically clarified, and we recommend considering the current evidence for potential secretory sources.

PGE2 is produced by all cell types in the body, including epithelial cells [32, 33], fibroblasts [34, 35], and infiltrating inflammatory cells [36–38]. Regarding the specific environment in OA, the sources of PGE2 are diverse, primarily involving MSC-derived cells and myeloid cells.

MSC-derived cells, including osteoblasts, osteocytes, and chondrocytes, are associated with PGE2 synthesis. COX2 expression in osteoblasts increases significantly, along with a decrease in trabecular bone and cortical bone, in OA mice post anterior cruciate ligament transection (ACLT) compared to that in the sham-operated mice [39], directly resulting in an increase in local PGE2 levels [39, 40]. Another study found that the specific deletion of COX2 in osteoblasts by crossbreeding COX2^fl/fl^ mice with Bglap-Cre mice ameliorates OA symptoms compared to COX2^fl/fl^ mice post-ACLT, with a decline in PGE2 levels [39]. In a spontaneous OA mouse model using the STR/ort mouse strain, an inbred substrain of STR/N mice, the knockout of COX2 in osteocytes attenuated the symptoms of OA, improving bone structure and moderating cartilage degeneration [41]. Additionally, PGE2 is secreted by chondrocytes in OA because proinflammatory cytokines, such as interleukin (IL)-1β, tumor necrosis factor, and IL-6, can induce COX2 expression and enhance PGE2 production [42]. Moreover, another study revealed that chondrocytes produced PGE2, while the use of caffeic acid phenethyl ester (CAPE) reduced the expression of COX2 in chondrocytes and the extracellular secretion of PGE2 in the cell culture supernatants [43].

In addition to MSC-derived cells, myeloid cells also participate in PGE2 synthesis. CD68^+^ macrophages function in PGE2 secretion through mechanisms involving divalent metal cations, which promote bone formation [44]. Although there are limited reports on PGE2 secretion, myeloid cells, such as T cells [45–47], macrophages [48], osteoclasts [49–51], and dendritic cells [52, 53], are important targets of PGE2 produced by MSC-derived cells to promote OA progression. We also observed that cementoblasts derived from MSCs around the teeth can secrete PGE2 under compressive pressure [54], indicating a complex array of cell sources for PGE2 secretion.

In summary, the effectiveness of NSAIDs implies that PGE2 acts as a potential therapeutic target, and secretory cells for PGE2 may be considered targets for treatment strategies. Although the side effects of NSAIDs cannot be ignored, understanding the mechanisms underlying the relationship between PGE2 and OA progression is crucial and may suggest novel treatments.

## 3. PGE2 Neuropathically Contributes to OA Pain

**Figure 1:**
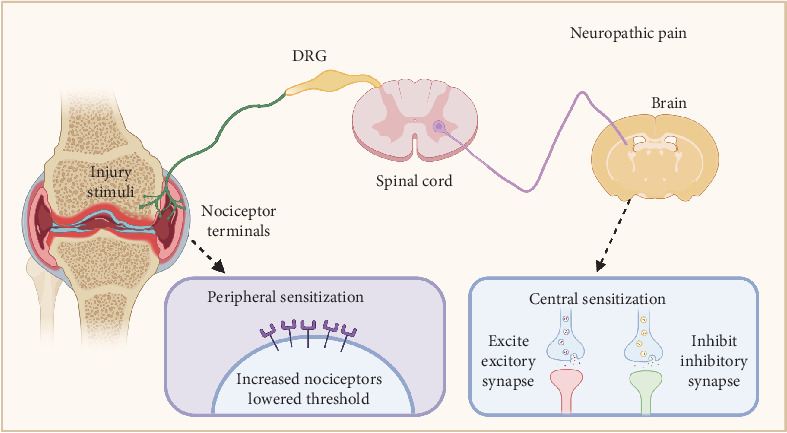
Mechanisms for neuropathic pain in OA. Nociceptor terminals are typically activated by stimuli in joints, and signals are transmitted along the DRG to the spinal cord and brain. Neuropathic pain in OA occurs when the number of nociceptors increases or the threshold decreases in peripheral sensitization. Central sensitization is mainly due to nervous system plasticity. DRG, dorsal root ganglia; OA, osteoarthritis. (Image created with BioRender.com).

The mechanisms underlying OA pain are complex [55] and have long been thought to be triggered by impaired structures and tissues or inflammatory pain, which can partly explain why pain is typically felt at the impaired joint. However, why patients with OA suffer severe joint pain, although the joint deformity and weight-bearing are mild, remains unclear [56]. Therefore, neuropathic mechanisms, including peripheral and central sensitization, may be involved in OA pain (Figure 1). Peripheral sensitization is characterized by the heightened responsiveness of peripheral nociceptors due to a lower threshold of local nerve excitation in response to injury, inflammation, or other nociceptive stimuli [57]. Central sensitization involves the excessive uploading of nociceptive signals and the downloading of inhibitory signals. Central sensitization arises from central nervous system plasticity, which produces pain by increasing spontaneous neuronal activity and widening receptive fields.

**Figure 2:**
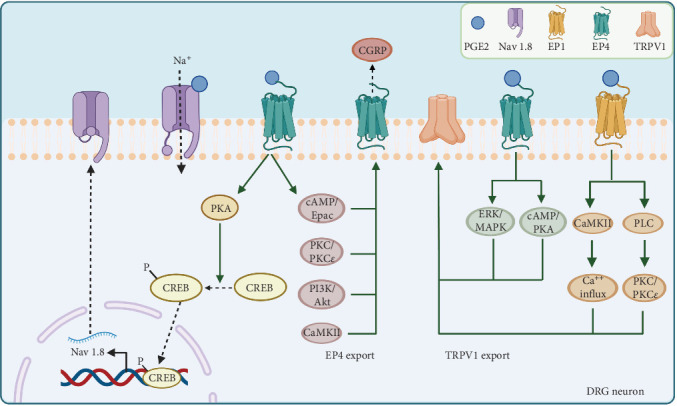
Novel pain mechanisms of PGE2 in peripheral sensitization in OA. (I) PGE2 activates EP4 and increases pCREB, which can bind to the Nav 1.8 promotor and enhance Nav 1.8 synthesis and externalization as well as Na+ currents. (II) EP4 activation by PGE2 recruits EP4 from the Golgi apparatus through the cAMP/PKA, PKC/PKCε, PI3K/AKT, and CaMKII signaling pathways, and externalized EP4 releases CGRP to cause pain. (III) TRPV1 externalization is promoted by EP1 and EP4 activated by PGE2 through the ERK/MAPK, cAMP/PKA, CaMKII, and PLC signaling pathways. Nav 1.8, voltage-gated sodium channel 1.8 C. cAMP, cyclic adenosine monophosphate; EP, E prostanoid; ERK, extracellular signal-regulated kinase; MAPK, mitogen-activated protein kinase; OA, osteoarthritis; pCREB, phosphorylated cAMP-responsive element binding protein; PGE2, prostaglandin E2; PKA, protein kinase A; TRPV1, transient receptor potential vanilloid-1 channel. (Image created with BioRender.com).

PGE2 directly sensitizes peripheral nociceptors, including the EP4 receptor [58, 59] and TRPV1 [60, 61 ], to cause pain. Nociceptor nerve terminals distributed in the joints are activated [62], and pain signals are transmitted along the axons to the nuclei located in the dorsal root ganglia (DRG), leading to the activation of second-order neurons [63, 64]. Ultimately, the ascending pathways activate the hypothalamus, thalamus, and higher somatosensory cortical centers to facilitate pain perception and recognition. However, recent speculation suggests that PGE2 also participates in neuropathic mechanisms, especially peripheral sensitization. Therefore, we summarize the recent advances in mechanisms of how PGE2 contributes to OA pain (Figure 2) through the voltage-gated sodium channel Nav 1.8, the externalization of the nociceptors like EP4 and TRPV1, leading to the increased cell surface density or activities of the receptor, and the combined effects between the receptors.

### 3.1. Nav 1.8

Nav 1.8, a voltage-gated sodium channel primarily expressed in DRG neurons and their nerve fibers, is highly relevant to pain signal transmission. PGE2 can promote the trafficking of Nav 1.8 through the protein kinase A (PKA) signaling pathway, which is mediated by an RRR motif in the first intracellular loop of Nav 1.8. Treatment with PGE2 in the DRG noticeably enhances channel density and Nav 1.8 currents, potentially inducing hyperalgesia [65]. Similar results were obtained when Nav 1.8-expressing neurons were chemo-genetically inhibited, efficiently blocking hyperalgesia and mechanical allodynia in early experimental OA in the knee joint [66]. Furthermore, the increased binding of phospho-cAMP response element-binding protein (pCREB) to the Nav 1.8 promoter markedly promoted the transcription of Nav 1.8, during which the EP4 in peripheral sensory nerves activated by PGE2 is the primary receptor [39]. Moreover, dampening PGE2 levels while modifying Nav 1.8 through the use of drugs conjugating TGF-β type I receptor kinase inhibitor (TβR1I) covalently with alendronate [67] effectively improves the structure of subchondral bones to alleviate OA pain [39]. Thus, this treatment pathway implies that balancing PGE2 and modifying Nav 1.8 may be a promising approach in the therapeutic management of OA pain.

### 3.2. EP4 Externalization

EP receptors participate in externalization to sensitize the DRG. Injection of 16,16-dimethyl PGE2, a stabilized PGE2 analog, can prolong tactile allodynia in a dose- and time-dependent manner. This effect appears to be due to the increased synthesis and anterograde axonal trafficking of EP4 at the axonal terminals of nociceptors [68]. Additionally, PGE2 exposure can recruit EP4 from the Golgi apparatus in the perinuclear region toward the cell surface in DRG neurons, which can be blocked by selective EP4 antagonists [69]. Two sequential treatments of DRG neurons with EP4 agonists resulted in a significant increase in the levels of intracellular cAMP compared with a single treatment, confirming that the externalized EP4 receptors induced by the first treatment were involved in nociceptor sensitization in the DRG by a positive feedback mechanism. Another study also confirmed that the sensitivity of externalized EP4 receptors is enhanced by measuring the release of pain peptides such as CGRP [70], which has been used as an indirect measurement for the activation of some pain receptors like TRPV1 [71] and EP4 [72, 73]. Moreover, PGE2 can directly enhance membrane tetrodotoxin-resistant voltage-gated Na^+^ currents [74] to sensitize DRG neurons associated with cAMP/PKA signaling [75, 76 ]. These studies suggest that agonist-stimulated EP4 externalization may be coupled with enhanced Na^+^ channel activity to elicit sensitizing effects.

### 3.3. TRPV1 Externalization

In addition to its direct effects, PGE2 activates TRPV1 externalization and enhances sensitization to capsaicin. Research has shown that when co-injected, PGE2 enhances the pain induced by capsaicin [77]. Other studies revealed that PGE2-induced thermal hyperalgesia was eliminated in TRPV1-knockout mice [78] or rats with unmyelinated C fiber neurons destroyed by capsaicin [79]. Moreover, PGE2 increases TRPV1 externalization by mobilizing TRPV1 stored in the Golgi complex while also increasing TRPV1 synthesis in DRG neurons to sustain persistent externalization. The key receptors for increased TRPV1 synthesis and externalization are EP1 and EP4, with involvement from the CaMKII, PLC, PKC, PKCε, cAMP/PKA, and ERK/MAPK signaling pathways [71].

### 3.4. Combined Effect of DRG Receptors

Furthermore, the co-localization and reaction between TRPV1 and EP receptors have also attracted attention. Owing to the upregulation of TRPV1 and EP4 levels and their enhanced functional activities both in the peripheral plantar skin and DRG of mice, the sensitization pain evoked by PGE2 is prolonged after pre-exposure to restraint stress compared to the vehicle controls [80]. Furthermore, the mechanical hypersensitivity can be reversed by the injection of the selective COX2 inhibitor NS-398, EP4 receptor antagonist L161,982, or TRPV1 antagonist capsazepine, suggesting that the COX2/PGE2/EP4 and TRPV1 signaling pathways contribute to prolonged sensitization pain. Additionally, selective EP4 antagonists significantly decrease TRPV1 currents and neuronal excitability in DRG neurons, reducing peripheral hypersensitivity and relieving pain [81]. These studies suggest that the functional interactions between PGE2 and TRPV1 are crucial for PGE2-induced peripheral sensitization.

## 4. PGE2 Participates in Joint Structure Alterations

In addition to its function in OA pain regulation, PGE2 is thought to be involved in bone homeostasis. A clinical test performed in patients with OA and normal controls showed a 50% increase in PGE2 concentration in the serum of patients with OA compared with controls and a significant enhancement in the lesion area compared with that in the nonlesion area [82]. However, whether PGE2 acts as a destructive or compensatory reparative factor in OA progression remains unclear. Here, we summarize the recent studies on the role of PGE2 in cartilage and subchondral bone alterations during OA progression.

### 4.1. Cartilage Alterations

PGE2 increases the production of chondrocyte MMP13 via EP4 in OA cartilage, inhibiting proteoglycan synthesis and stimulating matrix degradation [83]. Another study revealed that PGE2 activates EP4 to inhibit MMP-1 and MMP-13 production in OA chondrocytes by suppressing the MKK4-JNK MAP kinase-c-JUN pathway [84], presenting a contradictory finding. Both studies focused on PGE2 intervention in IL-1β-induced MMP expression and employed cartilage explant co-culture. However, the processing time was 13 h in one study compared to 24 h in the other. Moreover, one study used western blotting, and the other assessed MMP concentration in the culture supernatant. Different treatment times and measuring methods may have influenced the results. Furthermore, the different stages of articular cartilage obtained from the OA patients may have an influence on the results. One of the experiments focuses on the advanced OA patients undergoing the knee replacement surgery aged from 50 to 70 years, while another study involved 30 patients aged 72.0 ± 7.4 years. The different ages of patients and the lacking information for the accurate disease progress had impacts because of different stages of OA, leading to a discrepant microenvironment, which may indeed reverse the effects of PGE2. What's more, the distinguished gradient concentration of PGE2 and the effective concentration used in this two experiments also contributed to the opposed results. This concentration-dependent biphasic effects also reminded us that the effective PGE2 concentration in vivo needs more detection, and further studies are needed to explore the underlying mechanisms.

Despite EP4, EP2 also increases cyclic AMP levels via activation of adenylate cyclase. It has been found that EP2 selectively agonists prevented the cartilage degeneration at 2 weeks in rabbit traumatic degeneration model but had no significant improvements at 12 weeks, which indicated an early prevention fuctions of activating EP2 receptors [85]. Furthermore, another study focused on the chondral and osteochondral defects in rabbits found that selectively activating EP2 receptors enhanced the regeneration of the type II collagen-positive tissues with a physiological osteochondral boundary, which implied that EP2 effects for the regeneration of cartilage [86]. For the downstream mechanisms, it has been found that the simultaneous stimulation of EP2 and EP4 can cooperatively enhance proteoglycan accumulation and intracellular cAMP production in the rat primary chondrocytes, which contributed to the chondrocyte differentiation [87]. What's more, another study found that EP2 signaling downregulated MMP-13 mRNA expression via the cAMP–PKA pathway in human OA chondrocytes. Meanwhile, the activation of EP2 also inhibited several catabolic factors (MMP-1, MMP-3, MMP-13, ADAMTS5, IL-1β, and tumor necrosis factor alpha) and seemed to exert the anticatabolic effects [88], which may account for the recovery of the cartilage in vivo. Nevertheless, it has also been reported that the EP2 agonists have no effects on cell viability or proliferation capacity [88]. Meanwhile, another study found that PGE2 mainly activated EP2 receptors to suppress proteoglycan accumulation and synthesis as well as to suppress aggrecan gene expression through inhibiting PI3K/AKT pathway [89]. The contrast results of EP2 signaling on the catabolism and anabolism of cartilage may attributed to the different cell types in the obtained cartilage regions, OA or healthy sources, or the detective indicators. What's more, the different agonists used in the experiment may also influence the results for the nonunified concentration and the susceptible selectively activation.

### 4.2. Subchondral Bone Alterations

In addition to cartilage alterations, there are different opinions regarding the effect of PGE2 on subchondral bone. Elevated COX2 expression and PGE2 levels in the subchondral bone are associated with spontaneous OA, which can be improved using COX2 inhibitors in STR/N mice [41]. Another study on metabolic syndrome-associated OA discovered that the COX2-PGE2 axis may be the core mechanism underlying the observed subchondral bone alteration [50]. However, other studies [90–94] reported that NSAIDs significantly inhibit bone formation, indicating that PGE2 may play a significant role in inducing bone formation. For instance, PGE2 regulates the osteogenic differentiation of MSC by activating EP4 [95] and promoting osteogenesis. Several studies [40, 82, 96] have also demonstrated that a lack of EP4^+^ nerve fibers, the important target receptor of PGE2, can directly reduce bone density and bone formation capacity. The discrepancies between these studies suggest that the existence and normal function of PGE2 have a significant impact on the induction of bone alteration.

In summary, these contradictory results reveal the complex effects of PGE2 on OA progression, which is a breakthrough.

## 5. PGE2 Regulates Bone Alterations via EP4

**Figure 3:**
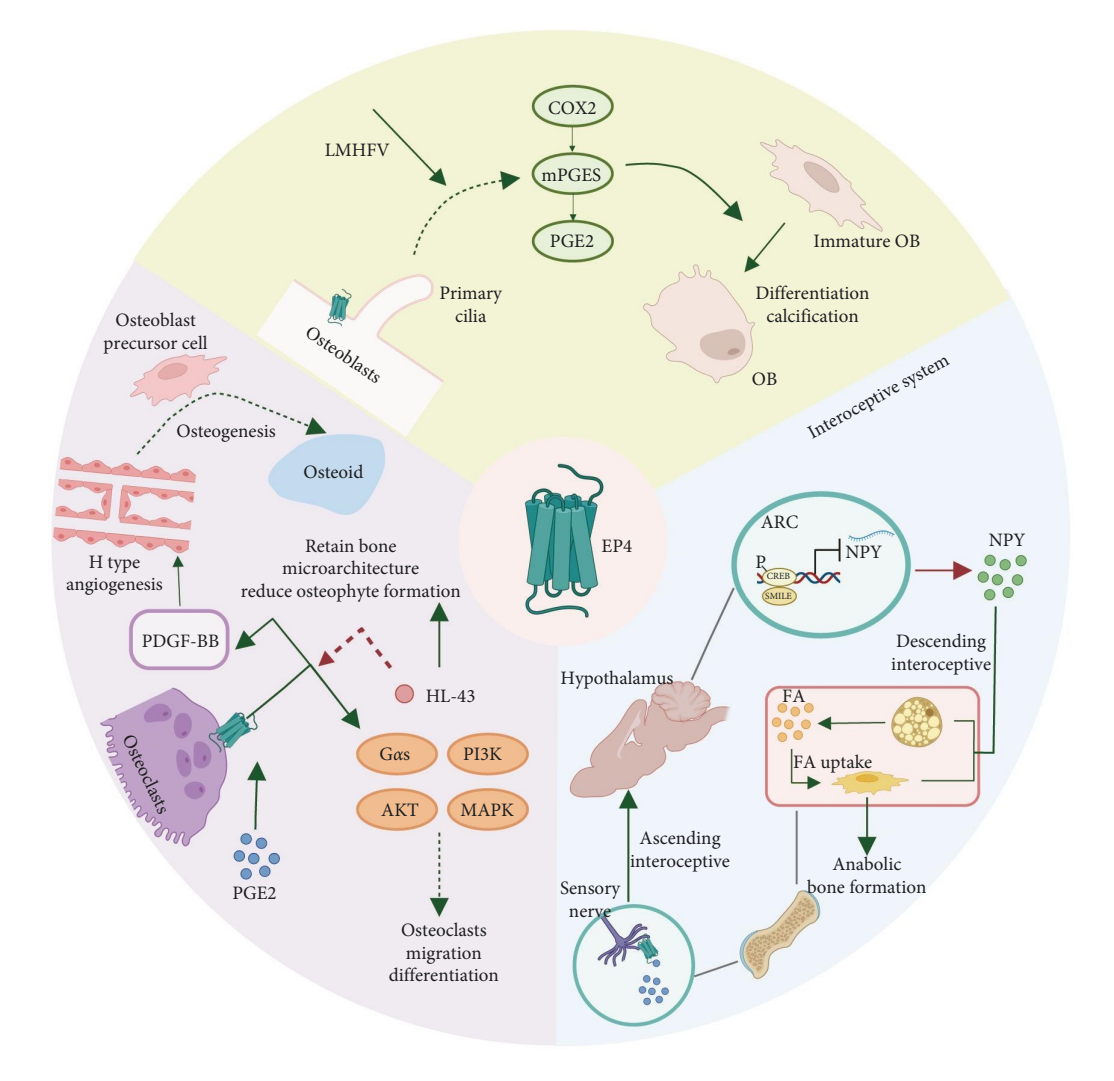
Mechanisms for bone alteration of EP4 in OA progression. (I) EP4 located at the base of primary cilia in osteoblasts can enhance COX-PGE2-EP4 signaling to induce osteogenesis under LMHFV. (II) EP4 expressed on osteoclasts can be activated by PGE2 and induce osteoclast migration and differentiation through the Gαs, PI3K, AKT, and MAPK signaling pathways and promoted angiogenesis to recruit osteoprogenitor cells to aggravate sclerosis, which can be blocked by HL-43 to alleviate OA progression. (III) EP4 expressed on sensory nerves can be activated and suppress NPY synthesis in the hypothalamus, which promote lipolysis of adipose tissue and oxidation of free fatty acids in osteoblasts thus inducing anabolic bone formation. (Image created with BioRender.com).

As widely recognized, EP4 is highly expressed in the bone [97], which has abundant sensory and sympathetic innervations and interacts with the central nervous system. It has been reported that EP receptors are expressed in sensory nerves [40], osteoblasts [98], and osteoclasts. PGE2 mainly acts on EP receptors to regulate bone homeostasis, particularly EP4, which is distributed in osteoblasts, osteoclasts [49], and interoceptive systems (Figure 3).

### 5.1. Osteoblasts

EP4 is expressed in osteoblasts, affecting mechanical osteogenesis. EP4 in osteoblasts is involved in bone homeostasis, together with primary cilia underlying low-magnitude high-frequency vibration (LMHFV), which has been reported to effectively induce osteogenesis [99]. LMHFV in osteoblasts increased the expression of EP4, the location of which was found to be based on primary cilia and modulated by them. The increased EP4 level participates in enhanced COX-PGE2-EP4 signaling to induce osteogenesis, which can be disrupted by an inhibitor of either factor. Therefore, the crosstalk between EP4 and primary cilia in osteoblasts offers a novel perspective on osteogenesis.

### 5.2. Osteoclasts

In addition to increasing osteogenesis, osteoclasts play an important role in the initiation and progression of OA. PGE2 activates EP2 and EP4 on osteoclasts to mediate their migration and osteoclastogenesis [49]. However, further research has shown that specifically knocking out EP4 can significantly attenuate OA progression and decrease osteophyte formation through TGF-β1 signaling [100]. What's more, the knock-out of EP4 on osteoclasts significantly reduced the secretion of VEGF-A, PDGF-BB, SLIT355, and angiogenin, which can regulate the production of H blood vessels. The angiogenesis contributed to the osteoprogenitor cells recruitments and further promoted to the remineralization and sclerosis, as well as the sensory nerve innervation.

Regarding downstream regulation, previous studies [101, 102] have suggested that PGE2/EP4 signaling may activate Gαs or β-arrestin in the kidney, gastrointestinal tract, and other tissues. The deletion of Gαs from osteoclast precursors impaired migration and differentiation to osteoclasts following PGE2 treatment by inducing cAMP production and downstream PKA signaling activation. Another study [49] indicated that cAMP, PI3K/AKT, and MAPK signaling altered the differentiation and migration of osteoclasts treated with PGE2.

### 5.3. Interoceptive System

Although EP4 in osteoblasts is effective in osteogenesis, knocking out EP4 in mouse osteoblasts did not affect bone formation [40]. Knocking out EP4 in CGRP + sensory nerves significantly reduced bone volume in adult mice and increased sympathetic tone through the central nervous system. Further studies revealed that PGE2 secreted by osteoblasts activated EP4 on sensory nerves and significantly increased CREB phosphorylation and CREB signaling activation in the hypothalamus. In turn, this directly suppressed sympathetic tone and enhanced osteoblastic activity by modifying the local microenvironment.

In addition to CREB signaling, neuropeptide Y (NPY) has been found to significantly impact bone metabolism downstream of PGE2. NPY is one of the most abundant neuropeptides in the hypothalamus and functions as an orexigenic peptide that mainly induces food intake [103]. PGE2/EP4 ascending interoceptive signaling balances bone formation and fat metabolism by regulating NPY expression in the hypothalamus [96]. PGE2 activates EP4 in the sensory nerves and enhances the expression of SMILE, a transcriptional repressor consisting of a heterodimer that binds to the NPY promoter and suppresses *NPY* gene transcription. The mechanism by which NPY influences bone metabolism mainly depends on the oxidation of free fatty acids in osteoblasts and the induction of anabolic bone formation.

Collectively, these data suggest that bone formation by osteoblasts and bone resorption by osteoclasts should be balanced to maintain the anatomical structure and normal function of the bone. In OA, overactivated osteoclasts accelerate bone damage, whereas overactivated osteoblasts can result in excessive ossification or the formation of osteophytes. Both processes contribute to OA progression at different stages, during which the interoceptive system plays a significant regulatory role.

## 6. PGE2 Regulates Bone Alterations via EP2

Similar to the circumstances of cartilage, EP2 has also been assumed to function in the bone alterations in OA. It has been found that the activation of EP2 along with EP4 enhanced osteoclastic differentiation of RAW264.7 cells in the presence of RANKL [104]. While another study found that the knockout of EP2 on osteoblasts decreased osteoclastogenesis of spleen cells when co-cultured [105]. These in vitro results provided the possibilities that EP2 may also affect the bone homeostasis in vivo. The studies [106, 107] focused on the bone fractures or bone mineral density have found that selectively activating EP2 receptors promoted osteonal remodeling and local lamellar bone formation as well as bone mineral content enhancement and bone strength parameters improvement. As for the OA microenvironment, one study used EP2^ko^ mice and found the osteoclast migration and differentiation aroused by PGE2 were significantly inhibited. However, EP2 deficiency had no effects on the OA progression when the researchers operated ACLT surgery on the EP2^ko^ mice to generate OA model. Thus, despite of the conceivable results in vitro, the definite evidence is still lacking for the effects of EP2 on bone alterations in OA microenvironment and needs much more explorations and more experiments to verify.

## 7. Future Perspectives for Therapy

As mentioned previously, subchondral bone undergoes different stages and pathological alterations. NSAIDs, along with COX2 inhibitors, have been the first-line medicine for patients with OA; however, the severe side effects of OA treatments are also attracting attention. Since the complex mechanisms by which PGE2 influences pain in OA and disease progression have been clarified, together with the spatiotemporal functional specificity of EP4, therapeutic strategies should be discussed in categories according to the stage of the disease (Table 1).

### 7.1. Therapeutic Strategies for the Early Stage of OA

Some perspectives suggest that the resorption of subchondral bone initiates pathological mechanisms since subchondral bone alteration precedes other changes. Hence, it is reasonable to suppress bone resorption, stimulate bone formation in the early stages of OA, and simultaneously reduce pain.

Since PGE2 mainly acts through the EP receptor, which appears to cause central sensitization-inducing pain and impair bone repair capacity, traditional strategies, such as COX2 inhibitors or EP4 antagonists, may relieve pain but hinder bone structure repair. Therefore, novel drug targets of PGE2 signaling may be more suitable downstream factors for achieving better curative effects. Following this approach, there are two chief perspectives: selectively inhibiting downstream factors that transmit pain signals while maintaining the osteogenesis of PGE2 or using COX2 inhibitors to relieve pain accompanied by the selective stimulation of bone repair.

#### 7.1.1. Selective Inhibition of Downstream Receptors That Transmit Pain Signals

As clarified earlier, Nav 1.8 participates in central sensitization in pain. Numerous animal studies have demonstrated that specifically knocking down Nav 1.8 effectively attenuates pain, indicating that blocking Nav 1.8 can be a promising treatment strategy. For instance, carvacrol, a predominant monoterpene in essential oils from many aromatic plants, has been reported to suppress the sodium currents of Nav 1.8 with an IC_50_ of ~ 300 μM; it is much more effective at inhibiting inactivated Nav 1.8 channels than resting channels in isolated rat DRG neurons, demonstrating its analgesic effects [111]. A-803467, a selective pore blocker, has been shown to have a preferential affinity for inactivated channels. It has been found to ameliorate inflammatory pain in a CFA rat model, neuropathic pain in a L5/L6 spinal nerve injury model, and chronic constriction injury in a sciatic nerve model at a dose range of 10–100 mg/kg [109, 119]. However, A-887826 was found to be more effective owing to its repetitive short depolarization in isolated murine DRG, with a half-blocking concentration of ~300 nM, exhibiting dose-dependent inhibition [108]. A clinical trial with VX-128, a highly selective Nav 1.8 inhibitor, indicated dose-dependent pain tolerance, suggesting its potential as a treatment [110]. Moreover, recent evidence suggests that the extracellular loop region may be an effective biological region for designing new drugs [112].

Concerning nociceptor externalization, EP4 also functions in bone remodeling; hence, it is more suitable for blocking TRPV1. Recent evidence showed that silencing TRPV1 can ameliorate neuropathic pain [120]. HCRG21, a TRPV1 blocker, has analgesic effects equivalent to those of NSAIDs [113]. Another study found that a recombinant peptide from the venom of the sea anemone *Heteractis crispa* had the same effect on pain relief. Moreover, SB366791, a selective TRPV1 antagonist, exhibits analgesic effects, exhibiting a 15-fold increase when combined with Phα1β, an N-type calcium channel blocker [115]. This suggests the potential for combined drug therapy, where recent studies have further clarified the underlying mechanisms through cryo-electron microscopy [114]. Additionally, the inhibition of downstream factors, including CaMKII [121–123], PI3K-A [116, 124, 125], and cAMP/PKA [126, 127], has been reported to be effective.

#### 7.1.2. Combined Use of Selective Bone Formation-Stimulating Factors With NSAIDs

The use of NSAIDs can lower the concentration of PGE2 and impair bone repair to some extent. Therefore, a combination of NSAIDs and bone repair stimulation strategies can ameliorate the side effects of bone destruction in the early stages of OA.

The EP4 antagonist HL-43 inhibits osteoclast differentiation, indicating that it may inhibit bone resorption [49]. Interestingly, HL-43 also induces articular regeneration by enhancing cartilage anabolism, suppressing catabolism, and promoting chondrocyte differentiation and extracellular matrix generation [128]. In addition, the oral administration of KAG-308 effectively suppresses chondrocyte hypertrophy and synovitis [129].

Furthermore, as effector molecules for activated EP4 receptors in bone repair, NPY and its receptor are also potential therapeutic targets. The high-affinity NPY Y1R inhibitor, BIBO3304, has been shown to rescue bone loss [96]. Another study demonstrated that γ-oryzanol can inhibit the synthesis of NPY and increase bone repair [117].

### 7.2. Therapeutic Strategies for the Terminal Stage of OA

At the terminal stage of OA, the activation of osteoclasts is suppressed, while osteoblasts are overactivated. This imbalance causes subchondral bone sclerosis and osteophyte formation, resulting in a thickened bone plate and decreased bone porosity. Hence, it is more appropriate to suppress osteogenesis and provide pain relief. The drugs mentioned below inhibit the concentration of PGE2 or directly inhibit its downstream signaling pathways, achieving this dual effect.

COX2 inhibitors are effective in decreasing abnormal concentrations of PGE2. Low-dose celecoxib has been found to maintain the physiological concentration of PGE2 in vivo [118]. Another study revealed that using celecoxib can ameliorate OA progression in the bone by controlling the concentration of PGE2 [82 ]. Additionally, EP4 antagonism can achieve the same goal by inhibiting downstream factors and suppressing excessive bone formation. As mentioned previously, HL-43 has demonstrated efficacy in relieving OA progression and attenuating osteophyte formation. However, this raises the question of how EP4 antagonists or agonists can selectively target receptors at specific sites, as the effect of EP4 activation on sensory nerves, osteoblasts, or osteoclasts may have different effector functions and require targeted therapy.

Although we hypothesize the potential mechanisms to intervene in the OA progression through EP4, but the exact alterations during the whole complex stages of OA are still worth verifying. The explorations of the main target cells or the vital responsive signaling in the early OA or the advanced OA make the therapy strategies more convincing and effective, during which the spontaneous OA model using EP4 agonists or antagonists in different OA stages may help. Furthermore, the transgenic animal model to intervene the receptors on different kinds of cells in different stages of OA can also verify the exact progression alterations. Therefore, further research on the structural differences or effective regions of these receptors is crucial to guiding novel therapeutic strategies.

## 8. Conclusion

In this review, we have summarized recent evidence for the critical role of PGE2 in pain sensitization and bone remodeling. By clarifying the underlying mechanisms, we can enhance our knowledge of OA progression, which can help develop more therapeutic strategies and pain relief across different stages of the disease. This will have significant implications for patients with OA and future treatment strategies.

## Figures and Tables

**Table 1:** Published studies on potential treatment drugs for osteoarthritis.

Disease	Function	Medicine	Regulatory details	Clinical stage	Reference
Early stage of OA	Analgesic effects ↑	A-887826	Sensitize Nav 1.8 and relieve use-dependent inhibition	Zoopery	[108]
			Through repetitive short depolarizations		
		A-803467	Block Nav 1.8 channels	Zoopery	[109]
		VX-128	Selectively inhibit Nav 1.8	Phase I clinical trial	[110]
		Carvacrol	Suppress the sodium currents of Nav 1.8	Cell experiment	[111]
		Extracellular loop region	A potential site for developing subtype-specific pore-blocking biologics	Cell experiment	[112]
		HCRG21	Fully blocked TRPV1	Zoopery	[113]
		SB366791	Bind to the vanilloid site as an allosteric hTRPV1 inhibitor	Cell experiment	[114]
		SB366791	Selectively antagonize TRPV1	Zoopery	[115]
		Combined with Phα1β			
		LY294002	Inhibit PI3K and regulate PI3K/AKT signaling pathway	Zoopery	[112]
	Articular repair↑	HL-43	Enhance cartilage anabolism suppress catabolism promote chondrocyte differentiation and extracellular matrix generation	Cell experiment	[114]
		KAG-308	Suppress chondrocyte hypertrophy and synovitis	Zoopery	[116]
	Bone repair ↑	BIBO3304	Inhibit NPY Y1R	Zoopery	[96 ]
		γ-oryzanol	Inhibit the synthesis of NPY	Cell experiment	[117]

Terminal stage of OA	Analgesic effects ↑	Celecoxib	Maintain the physiological concentration of PGE2 in vivo to induce bone repair and decrease bone porosity	Marketed	[82, 118]
	Excessive bone formation↓	HL-43	Target and antagonize EP4 receptors	Zoopery	[49]

## Data Availability

No data were used for the research described in the article.
